# The Tripartite Structure of Preparation Anxiety: Development and Validation of the Preparation Anxiety Scale

**DOI:** 10.3390/bs16091531

**Published:** 2026-08-31

**Authors:** Calen J. Horton, Christine M. Koscheka, Ashley M. Miller, Carolyn B. Murray

**Affiliations:** 1 Arkoda Research Group, Anchorage, AK 99508, USA; 2 Psychology Department, University of California, Riverside, CA 92521, USAashley.miller@ucr.edu (A.M.M.); carolyn.murray@ucr.edu (C.B.M.)

**Keywords:** preparation anxiety, performance anxiety, procrastination, self-efficacy, self-sabotage, self-handicapping, anxiety

## Abstract

Research suggests that anxiety predicts student performance, and that this relationship is mediated at least in part through self-defeating behavior. However, the nature of this relationship has yet to be fully understood. While substantial research has characterized the ways that anxiety affects student performance directly, at the point where it occurs (e.g., test anxiety, stage anxiety), less research has addressed the ways anxiety affects student performance indirectly, as students prepare for a performance. The first type is commonly referred to as performance anxiety. We refer to the second as preparation anxiety, and suggest that it is a necessary counterpoint, and that the relationship between anxiety and student outcomes cannot be fully understood without it. In the present study, we seek to characterize preparation anxiety, distinguishing it from performance anxiety and developing a scale to measure it: The Preparation Anxiety Scale (PAS). We report three studies, each conducted with undergraduate students at a large west-coast university: In the first study, focus groups were conducted to generate items representing the preparation anxiety construct. In the second study, exploratory factor analysis identified a 32-item, three-factor solution, with factors assessing affective nerves (PAS-AN), behavioral avoidance (PAS-BA), and cognitive agitation (PAS-CA). The scale displayed good test–retest reliability and demonstrated adequate convergent and discriminant validity. In the third study, confirmatory factor analysis supported the three-factor solution and bifactor analysis indicated a strong general factor, supporting the use of a summary score. Further analyses demonstrated both criterion and incremental validity, showing that the PAS and its subscales predict relevant behaviors (procrastination), correlates (academic self-efficacy), and outcomes (GPA) beyond other constructs. Additionally, regression analysis reveals a suppressor relationship: When the PAS-BA and PAS-AN subscales are controlled for, the PAS-CA subscale emerges as a negative predictor of procrastination and a positive predictor of academic self-efficacy.

## 1. Introduction

One of the most emblematic struggles of student life is the struggle against self-sabotaging behaviors. A substantive amount of research suggests that even when students care about academic outcomes, they often engage in counterproductive behaviors that undermine their chances of success. Covington’s self-worth theory (see Covington, 1992, 2009) suggests that this is driven by an underlying paradox; since an individual’s social standing and self-regard are often on the line in achievement situations, opportunities to achieve (or fail) are often experienced as a social threat. This sense of threat can, in turn, sabotage academic outcomes by encouraging compulsive avoidance or, alternatively, strategic bids to protect one’s self-image (e.g., Self-Handicapping; Jones & Berglas, 1978).

Accordingly, a substantive body of literature has demonstrated that anxiety and perceived threat are both related to poorer academic performance. In an early meta-analysis of the relationship, Seipp (1991) found a modest negative correlation between anxiety and achievement (*r* = −0.21). Similar numbers were found by later meta-analyses (e.g., Richardson et al., 2012; test anxiety *r* = −0.24). Research has also connected achievement to domain-specific forms of anxiety such as test anxiety (von der Embse et al., 2018), math anxiety (Barroso et al., 2021), and music performance anxiety (Steptoe, 1989).

The relationship between anxiety and self-defeating behaviors is similarly well-established: Anxiety and closely related constructs such as the personality trait of neuroticism are consistent predictors of trait procrastination (Steel, 2007).

In spite of this, it would be premature to conclude that we understand the relationship between anxiety and student performance, because there are two parts to the relationship, and one has been left largely uncharacterized. One half of the anxiety/performance relationship—the anxiety that students feel during a performance—has been treated as a formal construct for many decades. A substantive amount of research has been devoted to its variations, mechanisms, and to the moderating factors that influence its outcomes (Cassady & Johnson, 2002; Zeidner, 2014). By contrast, the other half—the anxiety that students feel during the period leading up to a performance, when they are preparing for it—has not been treated as a distinct construct in its own right. Little attempt has been made to formally model it: It is typically assessed using nonspecific measures such as state or trait anxiety, and when it has been studied, it has typically been subsumed into broader frameworks (e.g., procrastination, self-handicapping) as a covariate or predictor (e.g., Pychyl et al., 2000). Some research has been done on adjacent anxiety constructs such as anticipatory performance anxiety and writing anxiety, but these literatures are often isolated, overextended, or exhibit patterns of findings that point to the need for a measure addressing the broader preparatory context. To address this gap, in the present study we propose the development of a measure to assess anxiety in the broader context of academic projects, which we refer to as *preparation anxiety*. We begin by conducting a brief review of the literature, describing the need for a measure of preparation anxiety and defining the construct. We then detail a series of three studies in which we developed a measure of preparation anxiety in academic contexts based on the self-reported experience of students while preparing academic projects. Finally, we consider the implications of our findings as well as the limitations and possible future applications of our measure.

## 2. Anxiety: What Happens Before the Performance?

Preparation anxiety has received inconsistent treatment in the psychological literature: Based on our review, we have found three converging lines of research that have touched upon the construct of preparation anxiety. A substantive body of literature on self-defeating behavior has addressed it indirectly. A second body of literature has assessed adjacent constructs, generalizing from performance anxiety to preparation contexts through the mechanism of anticipation. A third line of research assessing writing anxiety is the closest analog of preparation anxiety that we have found, but the literature as developed is constrained both conceptually and functionally to the niche of research on L2 writing. While we cannot address every treatment of anxiety in preparation situations that appears in the literature, here we consider some of the most important instances we have found, as well as their limitations.

**Self-Handicapping.** Self-handicapping theory proposes that, in situations where they cannot be sure of success, students will sometimes fabricate obstacles in order to impede their own performance so that they can use those obstacles to obscure the reasons for their failure (Jones & Berglas, 1978). These obstacles can either be simple verbal excuses (e.g., claiming sickness) or actual physical obstacles (e.g., drinking instead of studying; see Hirt et al., 1991) but in either case they are not performance strategies, as they are, by definition, an obfuscation of performance rather than an attempt to improve it. Self-handicapping has been explicitly connected to uncertainty, a key component of anxiety (see Jones & Berglas, 1978; Prapavessis et al., 2003), and has also been connected to trait anxiety (Török et al., 2014) and contextualized forms of anxiety such as test anxiety (Schwinger et al., 2022).

The construct of self-handicapping was advanced by Jones and Berglas (1978) at a time when the cognitive revolution was in full swing and researchers favored conscious, agentic explanations for behavior. The pre-conscious and compulsive nature of emotions was not fully appreciated until later developments in cognitive psychology and neuroscience (see Lazarus, 1984) and largely for that reason early explanations of self-handicapping suggested that self-sabotage was a strategic and perhaps calculated behavior, enacted to manipulate information streams in a positive way regardless of whether one succeeded or failed (Jones & Berglas, 1978). Later research tempered this, finding evidence that it was more compulsive and directed at avoiding shame than accruing pride (Murray & Warden, 1992). Both of these explanations are possible, and in the absence of a serious endeavor to operationalize preparation anxiety and to study how it contributes to self-sabotage at the precise point where such behavior occurs, it is not possible to resolve the question of how common each motive for self-sabotage is. Researchers are left, instead, to infer explanations from adjacent findings rather than direct evidence.

**Procrastination.** The body of theory that procrastination research is built on draws largely from clinical accounts of procrastination transcribed prior to Solomon and Rothblum’s (1984) seminal work (see Horton, 2021, for a review of this history). Clinical literature established a theoretical link between fear of failure, anxious cognitions, and procrastination behavior that has been strongly supported at the level of self-report, with measures of procrastination and anxiety, as well as anxiety-adjacent constructs, consistently showing modest positive correlations (e.g., Pychyl et al., 2000; Solomon & Rothblum, 1984; see also Steel, 2007, for a meta-analysis).

However, anxiety has persistently shown a weaker relationship with behavioral delay (Horton, 2021), leading to a paradox: Anxiety is a stronger predictor of students identifying as procrastinators than it is of students actually putting off work. One plausible explanation for this is that anxiety produces a sense of pressure that some students resolve by procrastinating and others resolve by working early. This hypothesis is supported by a nascent body of literature on “precrastination” (see Rosenbaum et al., 2019; Sauerberger, 2019), and Horton (2021) has found that when a sample is divided into terciles by level of procrastination, the tercile with the lowest procrastination scores tends to show a negative association between anxiety and delay, working earlier, while the tercile with the highest procrastinator scores tends to show a positive association, working later. This analysis suggests that the relationship between anxiety and delay may be moderated by how students respond to anxiety as it occurs during the preparation phase. However, this possibility has been obscured by a tendency to overlook preparation anxiety in favor of broader and more distal measures such as state anxiety, trait anxiety, or test anxiety.

**Anticipatory Performance Anxiety.** One way of accounting for anxiety in preparation contexts is to treat it as an extension of performance. Generally, we have found that there are three approaches to this in the psychological literature, each with limitations.

The first approach, which is methodologically problematic, is to correlate trait-level measures of performance anxiety with measures of self-defeating behavior and then to imply (or simply state) that anticipation mediates the relationship (e.g., Aydemir, 2026; Hayat et al., 2024; Zeidner, 2014). This is problematic because cross-sectional, trait-level models can only establish that those who tend to endorse performance anxiety tend also to endorse that they engage in self-defeating behavior—a fact which is compatible with a range of causal models. Further, tests of the causal proposition suggest that it is false: A meta-analysis by Horton (2021) found that trait anxiety correlates far more strongly with trait procrastination than with actual work-delaying behavior, and a recent cross-lagged longitudinal study has found that procrastination at one time point predicts future test anxiety, but the reverse is not true (Wang, 2021).

A second and better approach has been the assessment of anticipatory performance anxiety both inside and outside the lab. A noteworthy body of research using the Trier Social Stress Test (TSST; Kirschbaum et al., 1993) assesses anticipatory performance anxiety by manufacturing a high-stakes, anxiety-inducing performance situation and studying the anxiety that people feel in the period shortly before it starts. Beyond the TSST literature, research on music performance anxiety has been underway for decades due to the impact of physiological anxiety symptoms on the fine motor skills needed for optimal music performance; some of this research has extended to the period of time preceding the test (e.g., Salmon et al., 1989; Ryan, 1998) and has found that some individuals peak during the performance while others peak before it. This literature does well at documenting the occurrence of anticipatory anxiety and its ties to maladaptive behavior but there are some conceptual barriers in generalizing it to preparation: the TSST paradigm compresses the anticipation phase into 20 min or less immediately prior to the feared event. Given the well-documented phenomenon of temporal discounting of future events (e.g., Steel, 2007), the findings associated with the TSST may not explain self-defeating behaviors that occur days or weeks prior to a performance. The findings from the music performance literature raise an additional question: If anxiety decreases for a substantial portion of the population after performance starts, it is unclear why performance should be treated as the conceptual explanation for the anxiety that precedes it.

A third approach, the Cognitive Behavioral Therapy (CBT) tradition, bears on this question. It offers a range of competing explanations for anxiety during preparation, few of which treat the performance as the center of interest. Ellis and Knaus (1977) treat the performance as little more than a trigger for maladaptive cognitions related to self-doubt, frustration, and rebellion. A more substantive bid at incorporating anticipation was developed by Clark and Wells (1995) to encompass social interaction (which broadly covers performance in front of people), positing that when high social-anxious individuals anticipate an upcoming interaction they engage in four maladaptive cognitions: They dwell on perceived past failures, create a negative emulation of how they think they will appear to others, over-monitor their bodily sensations, and then use the first three to make poor forecasts about how they will do (Clark & Wells, 1995; Hinrichsen, 1999).

These theories from the CBT literature offer a plausible mechanism by which performance might cause anticipatory anxiety. But again, there are gaps in the account that point to the need for a preparation anxiety construct. One is that there is a strong case that performance itself should not be considered the “parent construct” via which preparation is explained. It is clear that Clark and Wells’ (1995) model is built on evaluative threat, which is perhaps why anticipatory anxiety is associated with performance—performances are visible evaluations. However, evaluative threat is also present in preparatory contexts (like writing) that do not culminate in a performance. The implication is that performance and preparation might be two context-specific instantiations of a broader “evaluation anxiety.” At that point, it might be more parsimonious to simply treat the two contexts as different (though linked) manifestations of a broader anxiety, giving each context equal conceptual weight—rather than treating anxiety during preparation—in language, theory, and empirical modeling—as being dependent on performance.

Doing so may even detract by distracting from a second feature of preparation, which is that it has sources of anxiety that extend beyond the evaluative core it shares with performance: Task difficulty and complexity provide opportunity for frustration and self-doubt regardless of impending performance. The constant interruptions and competing priorities encourage clashing of personal goals, which can cause anxiety and distress (Gray et al., 2017). Deadline pressure introduces a dynamic, time-bound source of threat into preparation contexts (Steel, 2007) that, while sometimes present in performance (such as a test with a time limit), is still conceptually distinct from performance itself.

Our argument is not that anticipatory performance anxiety does not exert an effect during preparation—it clearly does. Rather, we argue that, whatever effect it exerts, it is better thought of as part of a dynamic system of anxieties that operate during preparation. This system—like performance anxiety itself—is sufficiently complex, and sufficiently common, to merit exploration rather than treating it as an extension of an established construct.

**Writing Anxiety.** Our research has turned up one construct that deals explicitly with the preparation phase, though it is isolated to a specific sub-domain of preparation. Research on writing anxiety addresses anxiety toward the act of writing itself. The literature on this, however, is fragmented in its treatment of anxiety. The primary measure used to assess writing anxiety is the Writing Apprehension Test (Daly & Miller, 1975), which contains few items that assess actual anxiety: It is primarily an attitudinal scale covering many aspects of writing, associating them with a variety of emotions. Later research (e.g., McKain, 1991) attempted to move toward a more comprehensive measure of anxiety itself, but the most successful of these measures has been the Second Language Writing Anxiety Inventory developed by Cheng (2004) to assess writing anxiety in L2 contexts. The development of these measures shows that the utility of studying preparation is recognized in at least one academic domain, but broader measures that assess preparation across more than one context have yet to be developed.

**Toward a unified construct.** These literatures have alluded to the existence of a shared phenomenon. Researchers who study self-handicapping frame it as ego defense in response to uncertainty (Jones & Berglas, 1978). Procrastination researchers incorporate it into theories of mood regulation or self-regulation failure (Pychyl et al., 2000). Anticipatory anxiety researchers try to explain it by treating it as an extension of performance. Some measures (e.g., foreign language writing anxiety) assess it in the domain of particular subjects. In each case, the literature suggests that preparation anxiety may be, in at least some cases, distinct from performance anxiety as it is classically conceived. To date, however, nobody has proposed to unify these threads into a single construct capable of capturing anxiety across the range of preparatory activities that constitute academic work, such as studying and writing. This is the gap that the construct of preparation anxiety is intended to fill.

## 3. Defining Preparation Anxiety

In the present study, we define preparation anxiety as a form of state anxiety that is experienced during preparation for evaluative academic tasks. As discussed, the nature of the situation—preparation—shapes state anxiety in ways that are unique to preparatory contexts, in a manner similar to the way that performance situations shape anxiety in ways unique to performance contexts. The contextualized nature of preparation anxiety means that it contains context-specific elicitors, targets, cognitions, and outcomes, and it is the combination of these that makes preparation anxiety worth consideration as a unique construct.

Consistent with the tripartite model of fear and anxiety (Lang, 1968; Martínez-Monteagudo et al., 2012), preparation anxiety is characterized by three broad components. The first is that it is an affective response, characterized by classic physiological manifestations of anxiety such as nervousness, dread, unease and tension, and also more somatically bound aspects such as muscle tension, physical complaints such as headaches, and an increase in heart rate, sweating, and blood pressure. The second is that the affective response, in turn, lends itself to a specific repertoire of behavioral responses, including avoidance (either alone or as part of an approach/avoidance cycle; see Roth & Cohen, 1986), distraction-seeking, self-soothing and/or self-stimulating behaviors, and difficulty engaging with work. Finally, the affective and behavioral dimensions are accompanied by anxiety-specific cognitions, which may include worry, racing thoughts, and stress about the preparation task itself.

Preparation anxiety is expected to overlap with several adjacent constructs, including general anxiety (state and trait), performance anxiety, and behavioral constructs such as procrastination. However, its context makes it distinct from these constructs in a few key ways, which we describe here.

**Performance Anxiety**. While preparation anxiety and performance anxiety are both manifestations of state anxiety, the unique contexts of each make different features of the anxiety response become salient. Performance anxiety, by definition, occurs in tightly constrained situations where avoidance is not possible. Therefore, behavioral manifestations of performance anxiety tend towards displacement behaviors meant to expel anxious energy in a situation where the desire to avoid must compete with the need to perform (see Troisi, 2002). Preparation anxiety tends to occur in loosely structured contexts where a person has the ability to leave. It is therefore expected that avoidance behaviors would be much more prevalent in preparation anxiety. Cognitions and physiological responses are similarly expected to be situation-specific. For example, impaired recall is important to performance constructs (see Eysenck et al., 2007) while the opposite, impaired encoding (either through physiological or attentional mechanisms), is likely to be more important in preparation contexts. Similarly, physiological mechanisms may be distracting in a performance context, which is short, but could become harmful to health and well-being in a preparation context, which can run for weeks.

**Procrastination**. Avoidant behaviors are part of preparation anxiety, but are conceptually distinct from procrastination. Preparation anxiety, as conceived, refers to micro-level avoidant behaviors that occur during preparation, which would encompass behaviors such as repeatedly leaving one’s chair, seeking distractions, or struggling to initiate a planned session. Procrastination, as typically treated in the literature, may sometimes include micro-behaviors but usually implies macro-level delay, shifting the entire preparation situation until late in the project cycle. The two constructs are likely to correlate highly but the macro/micro distinction between the two is useful: For example, macro avoidance (procrastination) can create situations where micro avoidance is greatly reduced because of last-minute deadline pressure. This effect is often prized by those who practice active procrastination, delaying projects intentionally to capitalize on the intense focus produced by deadline pressure (Chu & Choi, 2005).

With those distinctions being made, we now address the process of developing a measure of preparation anxiety for use in educational contexts.

## 4. Developing a Scale of Preparation Anxiety

At the outset of our research program, we determined that the best strategy would be a bottom-up approach that drew upon students’ personal experience. Because of the difficulty in observing preparation, there is little theoretical or empirical research to draw on for item construction. The few studies that do assess preparation on-site typically focus on a few pre-selected behaviors rather than on a holistic description of students’ preparation experiences. As such, the present research uses student interviews to address this gap by asking students about their experiences directly.

Across three studies, we developed and evaluated the Preparation Anxiety Scale (PAS). Study 1 used focus groups to identify relevant themes in the preparation process; a total of 66 items were identified for further analysis. Study 2 used exploratory factor analysis (EFA) to examine the factor structure of the items and narrow the item pool. The resulting 32-item measure (Koscheka, 2021) was then tested to establish reliability and validity. Study 3 used confirmatory factor analysis (CFA) to demonstrate the replicability of the factor structure, and further analyses were conducted to further establish incremental and discriminant validity of the measure and its subscales across multiple outcomes.

### 4.1. Study One

The purpose of the first study was to establish a preliminary item list for scale construction. To do this, we conducted focus group research to elicit descriptions of students’ experience (affective, behavioral, and cognitive) of the preparation phase.

#### 4.1.1. Method

**Participants.** Fifteen small group interviews were conducted with students recruited via the undergraduate psychology participant pool. Small group interviews ranged in size from one to three students; a total of 30 students (19 female, 11 male) participated. Participants were compensated with course credit for their participation.

**Procedure.** Upon registering for the study, students were given a short initial briefing and were asked to schedule an interview to take place at least one week after their registration. During that week, students were requested to spend time paying attention to their experience of doing homework and studying for tests. Upon arriving at the lab for their scheduled interview, students were provided with an informed consent briefing and, after signing, were interviewed by the lead experimenter. Interviews ranged in length from 10 min to 60 min (M = 25.6 min). During the interviews, students were presented with two scenarios—studying for a test and working on a project/paper—and were asked about their emotions in those situations, about the behaviors that they engaged in (and how those behaviors were related to their emotions) because of the feelings, and the behaviors that they engaged in to cope with the feelings. After describing these things generally, the students were also asked to imagine the most recent time they had been in those situations (i.e., test, paper) and to describe their emotions and behavior in those instances, as well as how close to the deadline they were when they actually started working. They were also asked their opinion about whether their behavior was compulsive, and were given a chance to add additional thoughts they considered relevant.

Interviews were recorded using a portable digital recorder and were transcribed by a team of trained research assistants. All transcripts were reviewed by a member of the research team to ensure accuracy. The resulting transcripts served as the raw data for item generation.

#### 4.1.2. Results and Discussion

In order to generate items, researchers proposed Likert-scale items that summarized themes described by the students during their interviews. Some students clearly indicated that they felt anxious during the preparation process and their items were kept as standard items for the item pool (e.g., “*I feel like my thoughts start racing*”). Other students stated plainly that they did not feel any sort of anxiety and did not struggle to work, and the items extracted from their responses were reverse-coded (e.g., “*I find it very easy to block out my emotions*”).

**Expert Review for Content Validity.** The list of proposed items was sent to a panel of three subject matter experts who had extensive experience with anxiety in educational settings. The panel reviewed the proposed items and evaluated them on their legibility and their pertinence to preparation situations. In addition, the panel offered notes and suggestions on item wording and flagged potential duplicate items for review and consolidation. Table 1 shows several example items that resulted from this process, as well as the raw statements the items were derived from.

### 4.2. Study Two

The purpose of the second study was to narrow the item pool generated in Study 1 as well as to establish and validate the factor structure of the resulting scale. We ran an exploratory factor analysis (EFA) on the 66 items generated in Study 1 and tested the resulting scale to establish test–retest reliability. We also correlated the scale with a range of well-studied covariates to establish convergent and discriminant validity, as well as to situate preparation anxiety inside the nomological network of constructs related to performance and self-sabotage.

#### 4.2.1. Method

**Participants.** To determine sample size for EFA, we relied on recommendations from MacCallum et al. (1999), which suggest that the adequacy of sample size depends on additional factors such as item-level communalities and the number of variables used to assess each factor, which are evaluated following the initial factor extraction. Rather than focusing on a specific item-to-participant ratio, we aimed simply to recruit a large sample size. For the present study, then, a total of 258 undergraduate students were recruited from a large Southern California university. Students were recruited from an Introductory Psychology subject pool via the institutional SONA system and were compensated with course credit for participation. Demographics for the sample can be seen in Table 2.

**Measures.** To assess preparation anxiety, we began with the 66 items retained from the first study. Supporting the idea that the items in Study 1 assessed a common construct, the initial Cronbach’s alpha value of the sixty-six items was α = 0.91.

We additionally included a battery of measures that assess constructs which are well-established in the literature on affect, self-sabotaging behaviors, and student performance. The full list of measures and their sources, along with Cronbach’s alpha values and relevant notations on item formatting, can be seen in Table 3. The purpose of the covariates was to provide evidence regarding convergent and discriminant validity as well as to situate preparation anxiety within the nomological network of related constructs in the psychological literature. Generally speaking, we anticipated that preparation anxiety would mirror other forms of anxiety in terms of its relationships to covariates: Being a form of anxiety, we anticipated that it would be positively correlated with other measures of negative emotion, positively correlated with self-defeating behaviors, and negatively correlated with a positive self-concept (e.g., high self-esteem, self-liking, self-competence). Correlations with these constructs, in the directions expected, were taken as general evidence of convergent validity. Of our covariates, the only two we did not expect to be related to preparation anxiety were the personality traits of agreeableness and openness, as these have shown weak or absent meta-analytic associations with anxiety and fear in other research (e.g., Kotov et al., 2010; Marengo et al., 2021), and low or absent correlations with these constructs were taken as general evidence of discriminant validity. All PAS items and covariates were assessed as traits except for the BDI-II, which assessed occurrence of symptoms over the past two weeks.

**Procedure.** The survey followed a two-wave design, with an initial large study and a smaller follow-up. The survey was presented using the Qualtrics survey platform and was approved by the local institutional review board. Upon recruitment, participants were provided with a link to the survey. After being presented with the informed consent briefing, participants indicated their consent to continue the study by entering their name on the consent page and proceeding with the survey. On average, the survey took approximately 40 min to complete, after which participants were debriefed.

After the debriefing for the first survey was complete, participants were given a link to the second part of the survey, made available to them one week later. The second survey was much smaller, consisting only of the initial pool of 66 preparation anxiety items, in order to assess test–retest reliability. The follow-up survey contained its own consent screen and debriefing, and took approximately five minutes to finish. The survey was made available for an entire week so that students could find a time that suited their schedule.

#### 4.2.2. Results

The analysis consisted of four phases: first, we prepared the dataset for analysis by screening and handling missing data; second, we assessed the data for suitability for EFA; third, we conducted iterative EFA to reduce the item pool and estimate the final factor solution; and fourth, we tested the resulting solution to establish reliability and validity. All analyses for the present study were conducted using IBM SPSS Statistics, version 27 (IBM Corp, 2020).

**Screening and Data Fidelity.** Six participants were removed from the analysis because they had failed to complete the full questionnaire, bringing the total to *N* = 252. The final dataset was screened for missingness; across all variables, the number of missing values did not exceed 1.6%, well within even conservative recommendations regarding missingness; Schafer (1999) argues that when missingness is below 5%, single imputation is likely to be accurate. Additionally, the results of Little’s (1988) MCAR test (χ^2^(33614, N = 252) = 2174.59, *p* = 1.0) suggested that the data was missing completely at random and that imputation was safe. As such, missing data for each variable was handled by imputing the mean value.

**Suitability for Factor Analysis.** The 66 proposed items for the PAS were tested to verify that they were suitable for EFA. First, they were screened for normality: Both skewness and kurtosis values for the 66 items were mostly below |1|, suggesting no meaningful departures from normality that would threaten factor-analytic results (Curran et al., 1996). Items were also screened for multicollinearity: Inter-item correlations did not exceed *r* = 0.80, and both the VIF values (1.6–5.3) and tolerances (0.19–0.62) were below the threshold for concern, suggesting that multicollinearity would not be an issue (Allison, 1999).

In addition to passing basic screening for normality and multicollinearity, we also tested the item pool on two additional criteria to verify its suitability for EFA. First, we tested to verify that items assess a common construct and covary to a degree that EFA is merited. Second, we tested to determine whether the patterns of covariance between items indicate the possibility of latent factors.

To address the first criterion, we started by computing Bartlett’s test of sphericity to determine whether the items shared sufficient covariance to merit EFA. Test results suggested this was the case; χ^2^(2145, N = 252) = 8198.36, *p* < 0.001. We then examined inter-item correlations to determine if any were off-construct: six items were removed because they did not correlate with any other item at a rate greater than *r* = 0.30, suggesting that they were poorly related to the rest of the set. As a final check, we computed Cronbach’s alpha for the remaining 60 items; the result (α = 0.91) suggested that the items assessed a common construct, although alpha values can be inflated by the size of the item pool (Cortina, 1993), so this was only intended as a preliminary check, for comparison to later reliability tests.

To address the second criterion, the KMO statistic was computed to assess whether the covariance among the items could potentially be explained by latent factors. The results of the KMO test (0.88) suggested the data was suited to factor analysis. As such, we proceeded with EFA.

**Item Reduction and Exploratory Factor Analysis.** For analysis, we used principal axis factoring (PAF) due to its suitability for assessing complex data where variables are assumed to reflect a latent structure (Costello & Osborne, 2005). We began by using an iterative PAF approach to reduce the item pool: PAF was conducted on the full set of 60 items, and items with factor loadings less than 0.40 were removed from the item pool; this process was repeated until all remaining items had factor loadings of 0.40 or higher, which reduced the item pool to 49 items from the initial 60. We then used a similar iterative approach to remove items with communalities less than 0.40, resulting in another 11 items being removed. The final pool of 38 items was used to estimate the factor solution.

To estimate an initial factor solution, we conducted Horn’s parallel analysis (Horn, 1965), which was implemented using O’Connor’s (2000) SPSS syntax. Parallel analysis compares the eigenvalues obtained from the correlation matrix of a set of observed items against the eigenvalues derived from a randomly generated set with the same sample size and number of variables. The central assumption is that factors reflecting a meaningful underlying structure in the data will produce eigenvalues that are greater than corresponding eigenvalues from random comparison data. When the observed eigenvalues are no longer significantly larger than the eigenvalues from random data, additional factors are no longer assumed to reflect meaningful latent structure. Eigenvalues for sequential solutions of the PAF exceed the comparison values up until the third solution, after which they are not significantly greater, suggesting that a three-factor solution was appropriate.

Several factor rotations were tested. Ultimately, promax rotation was selected on methodological grounds, as promax rotation accounts for intercorrelations between factors (Costello & Osborne, 2005), and it was anticipated that the factors of the PAS would be correlated with each other due to their representation of a common construct. This solution resulted in the removal of an additional six items due to insufficient factor loadings, bringing the scale down to 32 items. The final factor loadings can be seen in Table 4.

An examination of the three factors suggests that they are each internally coherent and represent different aspects of preparation anxiety. The first and largest factor consisted of 15 items (α = 0.92) describing common behaviors associated with anxiety during preparation, and was accordingly named *behavioral avoidance* (PAS-BA). The second factor consisted of nine items (α = 0.89) related to worry, uncertainty, and lack of focus, and was named *cognitive agitation* (PAS-CA). The third factor consisted of eight items (α = 0.87) describing anxious arousal and excessive emotional reactivity, and was named *affective nerves* (PAS-AN). Descriptive statistics and inter-factor correlations can be seen in Table 5.

**Reliability and Validity Testing.** To assess reliability, the scale was administered to participants a second time. A total of 188 students completed the second administration of the scale, which was made available one week after the initial survey. On average, students completed the second survey 7.8 days after the first. Test–retest reliability for the PAS was high, with strong T1/T2 correlations for the full scale (*r* = 0.81), the PAS-BA (*r* = 0.81), PAS-CA (*r* = 0.71) and the PAS-AN (*r =* 0.74). The intraclass correlation coefficient (ICC = 0.88) indicated high agreement between T1 and T2 assessments of preparation anxiety; by modern criteria, an ICC of 0.88 is considered good, and close to the threshold for excellent (see Koo & Li, 2016).

The correlation matrix between all study variables can be seen in Table 5. To assess convergent and discriminant validity, correlations between the PAS and our covariates were examined. All survey items were administered at the same point, raising the possibility that the correlations may have been boosted by common method variance (Podsakoff et al., 2003). To address this, we conducted a Harman’s single-factor test, conducting a principal components analysis on the 16 composites to see if a single factor could account for the majority of the variance between them (Podsakoff & Organ, 1986; Podsakoff et al., 2003). The first unrotated factor accounted for 41.8% of the variance, which suggested a modest amount of overlap between the measures but not majority dominance by a single factor. We discuss the implications of this in our Study 2 discussion.

The general pattern of results suggests that preparation anxiety and its subscales occupy a unique place in the network of concepts related to anxiety and self-defeating behavior. The PAS shared positive associations with all other fear constructs, including trait anxiety, test anxiety, and neuroticism. It was also positively associated with other negative emotions (depression, shame, and guilt), self-defeating behaviors (procrastination and self-handicapping) and intolerance of uncertainty. It was negatively associated with self-esteem, self-liking, self-competence, conscientiousness, and extraversion.

Our examination of the correlation matrix also suggests that the three subscales of the PAS share differential relationships with our covariates. The PAS-BA correlates markedly higher with our behavioral covariates, and is the subscale that correlates most strongly with self-handicapping (both the SHS and ASHS) and procrastination (PPS). It is also the strongest correlate of the PAS subscales with four of the five personality factors: conscientiousness, extraversion, agreeableness, and openness.

The PAS-CA, in contrast, is weakly related to most covariates. The only situation in which it is the strongest correlate is with the TOSCA-Guilt subscale. It is far more common for it to share the weakest relationship with covariates. It is markedly weaker than the PAS-BA or PAS-AN in predicting self-handicapping (SHS and ASHS), depression, self-concept (including self-esteem, self-liking, and self-competence) and is the weakest predictor of procrastination.

The PAS-AN is most strongly related to affective covariates. It is the strongest correlate of test anxiety and neuroticism by a modest margin, and is also the strongest correlate of shame. However, this pattern of strong relationships with affective correlates is not universal. The PAS-AN is not, for example, the strongest correlate of trait anxiety—the strength of its relationship with the STAI is nearly the same as that of the PAS-BA. Nor is it the strongest correlate of depression. Surprisingly, it is the strongest correlate of intolerance of uncertainty.

#### 4.2.3. Discussion

Our analysis yielded a three-factor solution accounting for 53.12% of the variance. The three factors were labeled according to the tripartite model of anxiety that they map onto—affective nerves (PAS-AN), behavioral avoidance (PAS-BA) and cognitive agitation (PAS-CA). In total, after removing items whose loadings or communalities were too low, the resulting scale consisted of 32 items. The intercorrelations between the factors (see Table 5) are sufficiently high to suggest that they measure a common construct, but are also low enough to indicate that they maintain distinctness as subscales, which suggests that the scale can be used to assess preparation as a global construct as well as to assess its components.

The subscales demonstrated adequate reliability. Internal consistency was excellent, with both the subscales and the overall scale having alpha values that were higher than 0.85. In addition, the PAS demonstrated strong test–retest reliability: Both the overall scale and the individual subscales correlated strongly with each other (all *r*’s greater than 0.70) across a one-week delay between administrations.

The pattern of correlations with covariates suggests that both the PAS and its subscales occupy a unique position within the nomological network of constructs that are used to understand and predict student performance. At the aggregate level, the PAS shares substantive overlap with trait anxiety, neuroticism, and self-esteem, which suggests that it is strongly connected to the standard cluster of anxiety-related traits. Its correlation with test anxiety, meanwhile, is weaker, suggesting that the two are related but distinct. It also shares substantial overlap with procrastination, confirming that it is related to classical self-defeating behaviors associated with student performance.

At the subscale level, the PAS-AN, PAS-BA, and PAS-CA show substantial divergence, suggesting that each assesses a unique component of preparation anxiety. Further, the pattern of correlations suggests that each subscale measures its stated aspect of preparation anxiety. The PAS-BA is more strongly correlated with behavioral measures such as procrastination and self-handicapping than the PAS-AN or PAS-CA subscales. The PAS-AN frequently emerges as the strongest correlate with other measures of anxiety or anxious temperament (though this pattern is not universal). The PAS-CA tends to be more modestly correlated with outcomes but emerges as the strongest predictor of one affective construct, guilt. While further analyses are needed, these initial results are promising and suggest that the subscales of the PAS may allow for fine-grained analyses that partition anxiety into its constituent components and allow for differential prediction of outcomes.

While the results of the EFA and the follow-up tests of reliability and validity are encouraging, there are a few limitations that should be noted. The first is the need for replication; while the factor solution found in the present analysis recapitulates the established tripartite structure of anxiety identified by Lang (1968; see also Martínez-Monteagudo et al., 2012), that does not imply that the factor solution itself is replicable. Further analyses in a different sample are required to validate the factor solution. Second, while our initial assessment of convergent and discriminant validity was promising, and the correlations of the subscales with outcomes were promising, we did not test for criterion validity (to ensure that the PAS predicts important academic outcomes) or incremental validity (to demonstrate that the PAS predicts outcomes and covariates over and above competing constructs such as state anxiety, trait anxiety, or test anxiety). Third, the PAS and the measures used to validate it were administered at a single time point, which raises the possibility that common method effects such as transient mood or similar item format may have inflated the relationship between the variables (Podsakoff et al., 2003). This is especially salient for variables that assess similar emotions to the anxiety measured by the PAS, such as the STAI, with which it shares a notably high correlation (r = 0.71). We conducted a Harman’s single-factor test (Harman, 1976) to assess the degree to which overlap between the PAS and our selected validation variables could be accounted for by a common factor, but this test is limited because the source of the common variance cannot be isolated and may be due to something besides method effects (Spector, 2006), and because Harman’s itself is not a conservative test (Podsakoff & Organ, 1986; Podsakoff et al., 2003). A preferred way to address this is to use methodological procedures such as separating the predictor (PAS) and the tested outcomes into different measurement phases: We do this in Study 3. Finally, in assessing the differential correlation between the PAS subscales and outcomes, we did not control for the intercorrelations between the scales. Study 3 addresses these limitations by conducting a confirmatory factor analysis (CFA) in a new sample, by testing the predictive power of the PAS against multiple academic outcomes, and by using regression analysis to account for intercorrelations between the PAS factors in predicting relevant outcomes and covariates.

### 4.3. Study Three

In Study Two, we established the three-factor solution for the PAS and provided preliminary support for its reliability and its validity. The purpose of Study Three was to replicate those findings and to answer questions about the relationship between the three factors. We tested the three-factor structure of the PAS using confirmatory factor analysis (CFA), estimating a series of competing models—a unidimensional model, a correlated three-factor model, and a symmetric bifactor model. We then conducted further validation tests: Whereas Study 2 made an initial pass to establish convergent and discriminant validity for the PAS, in Study 3 we addressed three finer points: the prediction of consequential outcomes over and above established anxiety variables (test anxiety, general anxiety, and neuroticism); the unique contributions of the three subscales; and a more focused test of discriminant validity, differentiating the PAS-BA from procrastination.

#### 4.3.1. Method

**Participants.** The participant pool consisted of *N* = 464 undergraduate students recruited as part of the Student Academic Success Study (SASS) at a four-year university in Southern California. Participants in the study were recruited using the institutional SONA system and were compensated with course credit. All study protocols were reviewed and approved by the IRB at the lead author’s institution. The study was a single cross-sectional survey that, due to length, was collected over two sessions. The PAS was administered during the latter session, which was taken by only *N* = 288 of the participants. Unless stated otherwise, all demographics and summary statistics reported from this point refer to the *N* = 288 sample. Demographics for this sample, along with comparisons to the full sample and also the sample from Study 2, can be seen in Table 2. The sample was skewed towards freshman and sophomore undergraduates, which is common with samples collected via institutional subject pool systems. We address the implications of this in the limitations section.

**Measures.** The present study makes use of the 32 PAS items that survived scale refinement in Study 2 in order to test the three PAS subscales—*affective nerves* (PAS-AN; 8 items), *behavioral avoidance* (PAS-BA; 15 items) and *cognitive agitation* (PAS-CA; 9 items). A more detailed description of the scale, including standardized loadings and precise alpha and omega values, can be seen in Table 4.

In addition to validating the factor structure for the PAS, we sought to demonstrate both criterion validity and incremental validity. To assess criterion validity we selected a range of criterion variables that assessed meaningful correlates of student well-being and success: We included measures of students’ self-reported GPA (both overall and major-specific), self-defeating behavior (the Pure Procrastination Scale), self-concept (the Academic Self-Efficacy Scale) and, reasoning that stress may have deleterious effects on physical well-being, a measure of student health (the Physical Health Questionnaire).

To assess incremental validity, we included measures of test anxiety (Westside Test Anxiety Scale), general anxiety (the anxiety subscale of the DASS-21) and neuroticism (from the BFI-2; Soto & John, 2017). Alpha values for these scales and our criteria variables can be seen in Table 3. All alpha values met or exceeded the minimum threshold (0.70) and were suitable for inclusion. The PAS and all covariates were trait-level measures, except the DASS and PHQ, which both assessed symptoms within the past week.

**Procedure**. After signing up via the institutional participant pool, participants were sent a link to a survey containing the study measures. The survey was administered in two sessions and participants were expected to complete the first session and return to complete the second within one week of the first. Most participants took approximately 40–60 min to complete the full battery of tests. The PAS and the DASS-Anxiety scale appeared in the second session; the rest appeared in the first session.

#### 4.3.2. Results

Analysis proceeded in three phases. First, we assessed the suitability of the data for CFA, conducting tests of missingness and normality and screening for common method variance. Second, we conducted CFA as an initial validation of the three-dimensional model in an independent sample; the tests conducted at this step supported the three-dimensional model established in Study 2 and supported the use of a bifactor model over both the unidimensional and three-factor correlated models. Third, we assessed the validity of the 32-item PAS. This included testing criterion validity at both the whole-scale and subscale levels, incremental validity of the PAS and its subscales above and beyond other anxiety measures, and discriminant validity of the PAS subscales relative to the closely related construct of procrastination.

**Suitability Analysis.** Due to the size of the survey, participants were asked to complete it across two sessions—after completing the first session, they were provided with a link to the second and asked to complete it within a week. Of the 464 participants, 176 did not complete the follow-up. To verify that this did not produce selection effects in our population, we ran a series of t-tests comparing survey completers (*n* = 288) to survey non-completers (*n* = 176) on all of our Study 3 variables and demographic markers. The two groups were not significantly different on any metric.

Next, we assessed the data for missingness and normality. For the 288 participants in the analysis, missing data for the PAS were minimal: complete data for all PAS items were available for 92.4% of participants. Average item-level missingness was 0.33%. Missingness was higher for criterion variables, but was concentrated in Major GPA (22.9%) and Overall GPA (10.1%), a pattern consistent with first-year students who have not declared their major. Since missingness was low and the data was 5-point Likert-style data we opted for the use of WLSMV with pairwise deletion to estimate the CFA models (which is appropriate for ordinal indicators with five categories or fewer; Rhemtulla et al., 2012; Flora & Curran, 2004) and used MLR as an additional robustness check. All PAS items met normality assumptions, with skewness ranging from −1.06 to 0.23 and kurtosis ranging from −1.14 to 0.48. Missing values for covariates and outcomes in correlation and regression models were handled using pairwise deletion.

The design of Study 3 partially mitigates the common-method concern that was raised in Study 2. Here, the PAS was administered in a follow-up session and was separated temporally from almost all other variables (with the exception of the DASS-Anxiety scale). A Harman’s single-factor test of the composites used in the study showed a first unrotated factor accounting for 45.0% of the variance, indicating that a single factor did not dominate the relationships among variables. Additionally, the DASS-Anxiety scale, administered contemporaneously with the PAS, only correlated modestly with the PAS composite (r = 0.43); roughly the same magnitude as the WTAS (r = 0.44) and notably lower than Neuroticism (r = 0.62), both of which were administered during a different session. In all, we judged the likelihood of common method bias to be low.

**Replication of PAS Dimensions.** Our first major goal was to replicate the three dimensions found in Study 2. Our second was to determine whether the 32 items of the PAS were essentially unidimensional (Rodriguez et al., 2015), and whether they could be aggregated into a single summary measure of Preparation Anxiety. To address these, we conducted CFA, estimating three models on the 32 PAS items—a unidimensional model, a correlated three-factor model, and a standard bifactor model. Models were estimated with WLSMV, appropriate for five-point ordinal measures like the PAS indicators (Rhemtulla et al., 2012; Flora & Curran, 2004). Relative to MLR estimation, WLSMV is more accurate at estimating factor loadings for ordinal data (Li, 2016). This consideration is of particular importance to our analysis because our indices of scale reliability for both our subscales and the general factor in our bifactor model are derived from the loadings. As a robustness check, we also estimated each model with MLR; all fit indices for each model, using both estimators, are reported in Table 6.

The correlated three-factor model was a better fit than the unidimensional model on every index (CFI = 0.886 vs. 0.791; RMSEA = 0.088 vs. 0.119; scaled Δχ^2^(3) = 278.20, *p* < 0.001), demonstrating the replicability of the three-factor solution from Study 2. However, the three-factor solution did not meet conventional thresholds for the incremental fit indices (CFI = 0.886, TLI = 0.877; Byrne, 1994) and its RMSEA exceeded the 0.08 standard (RMSEA = 0.088). The standardized residual index was acceptable (SRMR = 0.077). These decrements are unsurprising; as Kenny and McCoach (2003) have noted, incremental indices are often biased downward when a model contains many indicators even when the model is correctly specified. However, the implied model in a contextualized anxiety measure like the PAS is not simply three correlated constructs, but three distinct manifestations of an underlying emotional state: anxiety. As such, we also tested for the presence of a broader anxiety factor.

**Aggregation and Bifactor Analysis.** The model that is traditionally used to assess the presence of a more general construct—a hierarchical model with a second-order factor—cannot be tested when there are only three first-order factors. When tested, the resulting hierarchical model is just-identified and is statistically equivalent to a three-factor model with no superordinate construct, and cannot be informatively compared. A more useful approach is to use a standard bifactor model (Rodriguez et al., 2015) to partition variance at the item level into both a general factor and orthogonal specific factors. This allows for a determination of how much variance a general factor accounts for, as well as a way of establishing how distinct the sub-factors are. The bifactor model was a better fit to the data across all indices (CFI = 0.916, TLI = 0.903, RMSEA = 0.078, SRMR = 0.065) and cleared conventional thresholds, supporting the existence of a general dimension.

**Reliability and Dimensionality.** To assess the relative strength of the general factor and the specific sub-factors, we computed reliability and dimensionality indices and used them to evaluate the model (Rodriguez et al., 2016). Indices supported a strong general factor: Omega-hierarchical (ωH) indicated that the general preparation anxiety factor accounted for about 85% of total variance in PAS scores. Supporting indices (ECV, H, ω) converged on the same conclusion and can be seen in Table 7. Taken together, these indices support the aggregation of the 32 PAS items into a general PAS composite.

The specific factors retained little in terms of reliable variance after accounting for the general factor. Omega Hierarchical subscale values for the PAS-BA (ωHS = 0.19), PAS-CA (ωHS = 0.27) and PAS-AN (ωHS = 0.33) were modest, though it should be emphasized that this is the norm for scales in the social sciences, where subscales are used to represent aspects of broader psychological constructs. For comparisons to other common psychological measures, see Rodriguez et al. (2016). The omega hierarchical subscale values do not, themselves, weigh against the legitimacy of the subscales or their usefulness. However, they do bear on the way that the subscales should be used; we discuss this at length in the discussion section.

**Modification Indices.** Inspection of the fit metrics suggested that there was variance unaccounted for by the model. To investigate this, we looked at the modification indices, which flagged several item pairs as having substantial residual covariances. When dealing with modification indices, MacCallum et al. (1992) caution against freeing residuals liberally because it runs the risk of capitalizing on chance variations in the dataset. As such, we made the a priori decision to free residuals only if they exhibited a local justification for doing so, either exhibiting parallel wording and structure or being the result of a known method effect such as reverse scoring effects (Weijters et al., 2013). Based on these criteria, we freed four residual pairs. Two were reverse-coded (PAS-40 + PAS-58, MI = 46.4; PAS-40 + PAS-38, MI = 16.0). Two contained overlapping wording (PAS-14 + PAS-15, MI = 74.4; PAS-19 + PAS-36, MI = 16.5). We did not free other residuals even if they shared conceptual overlap in order to avoid the known pitfalls associated with post-hoc rationalization. With the four residuals freed, fit improved further (CFI = 0.930, TLI = 0.919, RMSEA = 0.071, SRMR = 0.061) but, importantly, even without these adjustments the bifactor model exhibited adequate fit, and freeing the residuals did not pass the higher threshold for good fit (CFI, TLI > 0.950). They should be viewed as a form of theory-driven optimization.

**Criterion Validity.** We first examined the full study correlation matrix (Table 8) to determine the relationship between the PAS (both total and subscale scores) and several relevant outcomes, including performance outcomes (overall and major GPA), health (the PHQ), behavior (procrastination), and self-concept (academic self-efficacy). The PAS-BA correlated with both overall GPA and major GPA; the PAS total score correlated with major GPA only, and the PAS-CA and PAS-AN did not correlate with either. The remaining variables—the PHQ (health), PPS (procrastination), and ASE (self-efficacy)—correlated with the PAS total score as well as with all three subscales. These correlations can be seen in Table 8.

An additional question remains—the subscales of the PAS are intercorrelated, and so a further test of criterion validity is to ask whether the subscales show differential relations with criterion variables when the intercorrelations between subscales are controlled for. We tested this by constructing regression models predicting each criterion from all three subscales in tandem (Table 9). Once we did, patterns emerged. The PAS-BA remained the sole predictor of both overall and major GPA, and the strength of the relationship increased after controlling for the PAS-AN and PAS-CA subscales. In the model predicting procrastination from the PAS subscales, the relationship between PAS-AN and the PPS score was no longer statistically significant; the PAS-BA was the strongest predictor, and the PAS-CA remained significant but the direction of the relationship reversed, indicating a suppression effect. The same pattern held for academic self-efficacy. Finally, in the model predicting health, only the PAS-AN was significant once the overlap between subscales was accounted for. The results of these models can be seen in Table 9. These subscale models improve substantially over the PAS summary score, accounting for a more substantial portion of the variance than the PAS total alone, and showing complex internal relationships between the components of anxiety.

**Incremental Validity.** As a final test, we assessed the incremental validity of the PAS subscales by using multiple regression to determine whether they predicted outcomes above three primary competitors—general anxiety (DASS), test anxiety (WTAS), and neuroticism (BFI-N). We used a hierarchical approach, first estimating two base models—one predicting a criterion variable from the PAS subscales alone, and one predicting the same criterion from its competitors. We then estimated four further models: the first three added a single competing variable on top of the PAS subscales, and the fourth added all three. We tracked R^2^ for each model and computed deltas to determine what the competitors added over and above the PAS, and what the PAS added over and above its competitors. The results can be seen in Table 10.

Generally, the PAS added value for our criterion variables. Two subscales—the PAS-BA and PAS-CA—were significant predictors of procrastination, and their predictive power was unaltered even when all three competing constructs were added into the model; in all, the PAS accounted for 17.4% of the variance beyond competitors. The same was true of academic self-efficacy: the PAS-BA and PAS-CA were significant predictors, their significance was unaltered by adding the competing anxiety constructs, and, in fact, when the competitors were added into the model, both test anxiety and neuroticism—which had been significant predictors by themselves—were no longer statistically significant.

The remaining criteria are more mixed but still generally support the contribution of the PAS. For physical health, the PAS-AN was a significant predictor and remained so even when test anxiety, general anxiety, or neuroticism were added into the model as single variables; it was only when all three were added together that the PAS-AN lost its predictive power, largely due to the combined action of the DASS-Anxiety measure and the BFI-N—neither alone was sufficient to reduce the PAS-AN to nonsignificance. The picture for GPA is more complex. The variance in GPA accounted for by the PAS subscales is modest (R^2^ = 0.045, or 4.5% of the variance) but this is comparable to other measures of anxiety in the psychological literature: Recent meta-analyses of the relationship between Test Anxiety and GPA find a modest correlation between the two (r = −0.17; von der Embse et al., 2018). Converted to a measure of variance accounted for, this is equal to about 2.8% of the variance, in the same range as the PAS total score, and lower than the combined subscales. The similarity in magnitude is evident in Table 10, where the models with the competing constructs of Test Anxiety, General Anxiety, and Neuroticism explain similar low-percentage amounts of variance as the PAS (R^2^ = 0.06 for Major GPA; R^2^ = 0.028 for Overall GPA). We evaluate the question of variance further in the Limitations section.

With that noted, the PAS-BA was a significant negative predictor of both overall GPA and major GPA, and adding competitors either individually or in combination was not sufficient to reduce it to nonsignificance. More interesting was that both the PAS-BA and WTAS were significant predictors of major GPA and remained that way even when entered into a regression model together, and even when controlling for other forms of anxiety and the remaining PAS subscales.

**Discriminant Validity.** An additional concern is raised by the high correlations between the PAS-BA and the PPS. While we have argued that the behaviors assessed by the PAS-BA are micro-avoidant behaviors—occurring in the moment during preparation, and conceptually different from procrastination—the high correlation between the two is a reminder that they need to be distinguished empirically, not just theoretically. To do this, we used CFA, comparing a unidimensional model—where PAS-BA and the PPS were constrained to load on the same factor—to a two-correlated-factor model where the items for each loaded on a factor specific to their measure. The fit metrics for the two-factor model (CFI = 0.891, TLI = 0.882, RMSEA = 0.097, SRMR = 0.080) were a substantive improvement over the unidimensional model (CFI = 0.805, TLI = 0.789, RMSEA = 0.130, SRMR = 0.107) and a scaled chi-square test of the difference supported that the two-factor was an improvement (scaled Δχ^2^ = 104.47, *p* < 0.001). As an additional test, Rönkkö and Cho (2022) recommend estimating the latent correlation (with factor variances fixed to 1) and examining the 95% confidence intervals—an upper bound under r = 0.80 is thought to indicate little or no problem. A test of the PPS and the PAS-BA (latent r = 0.687, 95% CI [0.620, 0.754]) again supports distinguishing between the two constructs.

#### 4.3.3. Discussion

The results of Study 3 confirm the three-factor structure identified in Study 2. Bifactor analysis establishes that the 32 PAS items can be aggregated into a general composite. Reliability indices at both the whole-scale and subscale level were high across both studies. Tests of criterion validity supported the relationship of the PAS and its subscales to several meaningful outcomes, including procrastination, academic self-efficacy, physical health, and GPA. Importantly, the relationship between the subscales and our criteria, when controlling for intercorrelations between the subscales using multiple regression, showed that each scale had unique relationships with other variables. The PAS-BA was a strong predictor of behavior, self-concept, and performance outcomes, and was the strongest of the three subscales. The PAS-AN shared a unique relationship with physical health. The PAS-CA, meanwhile, exhibited suppression effects, emerging as a negative predictor of procrastination and a positive predictor of academic self-efficacy once the PAS-BA and PAS-AN subscales were controlled for. Tests of incremental validity further demonstrated that the PAS predicted outcome variables above and beyond other conceptually related measures such as test anxiety, general anxiety, and neuroticism, and in some cases even when all three were controlled for. A follow-up CFA confirmed that the PAS-BA was empirically separable from procrastination. Taken together, the results of Study 2 and Study 3 offer strong support for the reliability and validity of the PAS and its subscales.

## 5. General Discussion

Existing measures of anxiety in the context of student performance conceptualize anxiety either as a context-free construct (e.g., state anxiety, trait anxiety, neuroticism) or in relation to high-stakes performance situations (e.g., test anxiety). The present study proposes that anxiety during preparation situations differs from these other forms of anxiety due to its unique context. Across three studies, we developed a measure of preparation anxiety—the PAS—to assess this construct in academic settings. In Study 1, we generated an initial pool of items by conducting short interviews with groups of students to inquire about their experience of preparation anxiety. In Study 2, we used EFA to explore the factor structure of the resulting items, concluding that a three-factor solution was the best fit for the data, and conducting initial tests of reliability and validity. In Study 3, we verified the factor structure using CFA and conducted follow-up tests to establish criterion validity and incremental validity. Here, we take time to review what we consider to be the most substantive findings of this project and consider their implications for the literature as well as how they might be implemented.

**Using the PAS.** The PAS fills a meaningful gap in student performance research. Its items were derived from students’ self-reports of their experience with preparation for common academic challenges. When tested using EFA, those items formed a three-factor structure representing a context-specific instantiation of Lang’s (1968) tripartite model of anxiety, capturing affect, behavior, and cognition. The three-factor structure showed high test–retest reliability, high internal consistency, and replicated across two separate samples. Evidence for the validity of the PAS has been demonstrated cumulatively across studies: Study 1 established content validity by deriving PAS items directly from student descriptions. Study 2 established convergent and discriminant validity by situating the PAS in the network of psychological constructs used to understand student performance. Study 3 demonstrated criterion validity by predicting consequential outcomes (behavior, performance, and health) at the whole-scale and subscale level. It also demonstrated incremental validity, predicting relevant outcomes above and beyond other anxiety variables, including test anxiety, general anxiety, and neuroticism. It is not perfect—a notable weakness is that the PAS total score, subsuming between-subscale suppressor effects, sometimes underperforms its own subscales. But the cumulative pattern of findings supports its reliability, its validity, and its utility.

***The PAS in Practice.*** One of the primary questions of interest regarding the PAS is how it should be applied at the practical level to facilitate student growth. In this regard, we think the benefit of the PAS is that it has potential for differentiating the “locus” of anxiety in a way that has not been done before. In conjunction with measures of performance anxiety and global anxiety, it should be possible—with the appropriate development of the construct—to identify where the “break point” is for students who struggle with anxiety: Does it affect them more when they are preparing or performing? Interventions can be targeted accordingly.

In terms of the technical aspects of measure use, we have two suggestions. The first is a matter of its use in the assessment of individuals: Our bifactor analysis suggests that when interpreting individuals’ scores the appropriate use of the PAS is to combine the scores into an aggregate measure of the general factor: Administering the three subscales and trying to interpret the results for individuals is imprecise because it is unknown how much of a single score is attributable to the general factor rather than to subscale-specific variance. In such instances, the general PAS score is likely to be the safest.

This is, however, a general principle: Our second suggestion is more specific, and pertains to the PAS subscales. While it is difficult to determine, for an individual case, what proportion of a score is attributable to the general factor and what proportion is attributable to the specific factor, there are some uses where this may not be needed. When dealing with an outcome where the general and specific factors are additive—that is, where they both contribute in the same direction to predicting an outcome—a PAS subscale score may be a more useful measure than the PAS Total. This is evident from an examination of our correlation matrices; in measures of performance (GPA) or self-defeating behavior (PPS, SHS, ASHS), the PAS-BA was a stronger predictor than the PAS Total score. Similarly, for physical health, the PAS-AN was a better predictor. However, we suggest this with caution, as more research is needed to establish when a subscale should be preferred to the total score. The general principle should be to rely on subscales for individual assessment only when additivity has been established. We recommend that researchers and practitioners consider this use case and inquire into it further.

***The PAS in Research.*** The results of our analysis suggest two complementary ways that the PAS can be used. First, the 32 items of the PAS can be aggregated into a single global index of trait-level preparation anxiety. The results of our bifactor analysis (ωH = 0.85, ECV = 0.69) show that the general factor comprises the majority of the common variance, justifying aggregation (Rodriguez et al., 2016). In order to compute the PAS composite, we recommend averaging at the item level (i.e., all 32 raw items) rather than the subscale level. While the composites produced by the two approaches are strongly correlated, item-level averaging is supported by the logic of the bifactor model. Subscale-first averaging, while not countermanded by our results, was not tested in the present analysis. The resulting score should be interpreted as a tripartite construct, including behavior, rather than as a purely affective/cognitive experience.

Second, the PAS items can be gainfully aggregated into the three PAS subscales—the PAS-BA, PAS-CA, and PAS-AN. The most informative use of these subscales is to enter them together into a single regression model; doing so makes it possible to partition their contributions to an outcome, revealing complex and occasionally contradictory relationships between affect, behavior, and cognition. While the subscales can be used alone, raw subscale or total scores—and the relationships they share with other constructs—should be read with the understanding that they reflect the general construct of preparation anxiety first, and the specific variance of the subscale second. This is a common feature of multidimensional measures that assess dimensions of a psychological construct; almost by definition, they index a strong general factor (Rodriguez et al., 2016). For this reason, when the goal is to predict an outcome from the facets, or to distinguish between them for some other use, we recommend using them in a manner that controls for their intercorrelations (i.e., multiple regression). This is especially important in light of the suppression effects uncovered in this study: Such effects imply that, for some outcomes, the subscales of the PAS are in tension with each other.

**Tripartite Anxiety and Differential Prediction.** The results of our incremental validity tests suggest that the PAS accounts for variance above and beyond other anxiety measures. Results for the PAS subscales in particular suggest that the tripartite model in the context of preparation is generative. Each subscale appears to not only predict more variance but also to serve a qualitatively different role; the PAS-BA is an especially strong (and often negative) predictor of outcomes related to performance and behavior. The PAS-AN emerges as a robust predictor of physical health—a distal outcome that is not directly related to academic performance but that is nonetheless important for student success. The PAS-CA, after controlling for the other subdimensions, appears to predict good behavior (at least for these variables). Put simply, the subscales do not just explain more variance; they do different things.

The PAS is one of a small number of anxiety measures in academic contexts that uses Lang’s (1968) tripartite model as the underlying frame for its factor structure. McKain (1991) and Cheng (2004) have both explicitly assessed affect, behavior and cognition in the specific subdomain of writing anxiety; our structure is congruent with their models and goes beyond them in terms of domain generality. Outside of preparation constructs, there are a few other measures: a small number of test anxiety measures contain behavioral dimensions, though the majority of these assess test anxiety in children or adolescents (e.g., Wren & Benson, 2004; Lowe, 2018). Some similar efforts have been made regarding the separate but related construct of speech performance anxiety, but the results of these have been mixed; Bartholomay and Houlihan (2016) attempted to use items representing the tripartite model to create a measure of public speaking anxiety but their measure resolved into a two-dimensional framework assessing cognition and physiology.

This latter case is not unusual: Though scales with explicit tripartite structures are not common, behavioral items appear regularly in contextualized anxiety measures even when they do not wind up occupying a unique behavioral dimension. The Sarason Test Anxiety Inventory (Sarason, 1980), for example, contains items that assess cramming, studying, motivational freezing, and eating. The Westside Test Anxiety Scale used in the present study (Driscoll, 2007) contains items assessing attentional control and avoidance. In part, this is because of the nature of context-specific anxiety measurement: Scales are developed to assess anxiety when it is a problem; therefore, they often wind up including statements regarding the problem in their item text.

Importantly, however, this does not mean that the influence of those items goes away. Rather than disappearing, items related to maladaptive and avoidant behavior become embedded in the other subdimensions of the measure, making them difficult to partial out in situations where researchers want to find out how affect and cognition behave separately from self-sabotage. This is particularly important because the interrelationships between affect, behavior, and cognition are not settled among anxiety researchers: Many researchers emphasize the separability of behavior from anxious and fearful emotion (e.g., LeDoux & Hofmann, 2018), while others include it in measures of anxiety because of its diagnostic value (Beesdo-Baum et al., 2012). While remaining neutral on this conflict, we note that in either case the resolution, in psychometrics, is not to allow behavior to linger as a hidden confound: by measuring it, the PAS allows it to be controlled for statistically, which makes it a more useful measure to both sides of the debate.

**Suppression Effects.** With the above in mind, the results of the present study are informative, at least within the specific context of student academic preparation. The PAS-BA, PAS-CA, and PAS-AN not only exhibit effects unique to each subscale; there is also a general pattern in their results, which is that the PAS-BA is, across all of our tested variables, in conflict with the PAS-CA and sometimes with the PAS-AN. These findings are congruent with the general pattern predicted by the literature, where the defining feature of anxiety is internal conflict (McNaughton & Corr, 2004). This may sound counterintuitive because the common way of defining anxiety is to say that it is a response to ambiguous threat, but that explanation glides over a cognitive architecture defined by conflict: When threat is not ambiguous, an organism is impelled to run and no other considerations intrude. When threat is ambiguous, the organism is torn between two conflicting needs: to avoid and to monitor. As McNaughton and Corr have noted, this appears to generalize beyond simple “ambiguous threat” scenarios and can be seen at least partially as a byproduct of conflicting goals.

Given that our results are cross-sectional, we read this to mean that at the population level the subset of students who experience preparation anxiety exhibit similar motivational arousal but some succeed in resolving the conflict adaptively while others capitulate to fear-based avoidance. Those who avoid fare worse, overall, than those who resolve their fear through other methods. This echoes the general findings of the coping literature (Lazarus & Folkman, 1984) as well as research on procrastination that finds some students respond to anxiety by working earlier (Rosenbaum et al., 2019; Horton, 2021). It also reflects the duality described by Covington (1992), where highly important situations produce both a desire for success and a fear of failure, as well as the classic interpretation of anxiety as being partially adaptive (Yerkes & Dodson, 1908). We suggest that one strength of the PAS is that, in modeling anxious avoidance and allowing for it to be statistically controlled, it facilitates research into the adaptive properties of anxiety.

## 6. Limitations

**Self-Reported, Trait-Level Assessment.** One of the primary limitations of this study is the use of self-report. We use the term limitation here in the truest sense; it does not imply a sub-optimal study design (since the purpose of this study was to develop a self-report scale) but instead is intended as a comment on the paradigm of self-report. We addressed a primary concern regarding self-report—common method variance—both structurally (by separating measures across two data collection sessions) and statistically (through the use of Harman’s single-factor test). Method variance arguments, however, are just smaller instantiations of a larger concern, which is that self-report does not always converge with direct measures of physiology or behavior. Accordingly, the PAS should be considered a measure of students’ holistic, trait-level self-evaluation of anxiety felt during preparation, and consideration should be given to how it is applied; if administered as a broad trait-level inquiry, the results should not be taken as evidence of state-level physiological arousal felt during particular episodes. If administered as a measure of a specific episode, it should be interpreted with caution and should be re-validated for that use, as the current validation is of the PAS as a trait-level measure. Its use for detecting specific state-level events has not yet been validated and must be tested. We do not believe that this weighs against the present analysis, but we do believe that it provides an important reminder that self-report, while useful, is only one form of psychological data (see Funder, 2019), and future research should use this measure as a starting point, identifying people who report maladaptive anxiety during preparation to facilitate further exploration with real-time measures of physiology, cognition, and behavior, as each unfolds in vivo.

**Sample and scope.** The PAS was developed using content drawn from undergraduates describing how they study and write. The particular recruitment population—introductory psychology students—means that freshmen and sophomores are overrepresented (see Table 2). Accordingly, our scale should be most applicable to students of the same level performing similar activities. Similarly, the precise descriptive statistics and demographic characteristics reported here may not represent upper-division, other-institution, or non-undergraduate (e.g., graduate or professional) populations, nor populations outside academia. The sample did, however, consist of students from a wide range of institutional disciplines, as the introductory courses were attended by students from a variety of majors. Studies 2 and 3 quantified student majors specifically, finding substantial representation from both STEM and HASS students. The majors for students in Study 1 were not recorded, but they were drawn from the same population through the same recruitment method.

Self-reported GPA in the present study was range-restricted (M ≈ 3.3, SD ≈ 0.5), which likely attenuates the criterion correlations; samples with a broader range of GPA may accordingly find stronger effects. These, however, are boundaries of the sample, not the construct: The focus on academic preparation was by design, and the results of our analyses support its use in educational contexts. We expect that the measure will also be useful beyond academic settings, but we recommend that, if researchers wish to employ variants of the measure in populations that are substantially different from the one here, they should re-verify its psychometric properties instead of proceeding uncritically.

**Specific-factor reliability.** Because of the strength of the general preparation anxiety factor, the subscales of the PAS retain only modest variance after the general factor is controlled for (ωHS = 0.19–0.33). We note this for the purposes of being thorough, but we also suggest that it should be considered in light of theory and also the substantive findings of the current study. First, it is expected that facets of a single psychological construct should share a strong general factor—two anxiety subscales that did not would be a much greater cause for concern. In support of that, low omega-hierarchical-subscale values are also common in the literature. The ones found for the PAS-BA, PAS-CA, and PAS-AN exceed those of many measures surveyed by Rodriguez et al. (2016), meaning that they are comparable to or better than many well-established measures.

Just as important, the low omega hierarchical subscale values should be contextualized in light of the substantive findings from the discriminant validity analyses. The PAS-BA and PAS-CA, especially, show patterns of results that are consistent with known effects in the literature and that appear across multiple variables. Low reliability of the specific variance attributed to the subscales should weigh against the likelihood of finding such effects by attenuating the subscale-specific signal. That consistent and theoretically aligned results were found anyway suggests that the signal carried by the subscale-specific variance is strong. Once again, this supports the recommended use of the measure: the three subscales should be used together.

**Low criterion variance.** While the PAS and its subscales account for a substantive amount of variance in self-report outcomes such as procrastination (R^2^ = 0.411), academic self-efficacy (R^2^ = 0.183), and self-reported physical health (R^2^ = 0.164), the amount of variance accounted for in GPA is comparably low, being only 4.5% of the total for overall GPA and 4.3% for major GPA. While acknowledging this feature of our analysis, we think that its implications for the PAS are positive. Funder and Ozer (2019) have noted that judging models in terms of variance accounted for can be misleading because variance is a squared metric. Effectively this means the first few percent of the variance accounted for are weighted heavily in terms of real-world impact: A variable that accounts for only 1% of the variance in an outcome, when converted into a normal linear correlation by taking the square root, is equivalent to *r* = 0.10, roughly equivalent to the correlation between taking an antihistamine and reduced sneezing (r = 0.11; see Meyer et al., 2001).

This property of variance can be applied to regression models as well. Our model predicting overall GPA from the three PAS subscales accounts for 4.5% of the variance, which translates to a multiple correlation of R = 0.21 between the model prediction and the outcome variable. The standard bivariate correlation between the PAS-BA (the strongest subscale-level predictor) and overall GPA is r = −0.17. These correlations are comparable in magnitude to many other common psychological predictors of GPA, including Test Anxiety (r = −0.17; von der Embse et al., 2018) and conscientiousness; the latter, included in our correlation matrix in Table 8, correlates with overall GPA at r = 0.18, the same magnitude as the PAS-BA and Test Anxiety. Thus, while the correlations are modest, this is common when predicting GPA from self-report measures. We therefore argue that the most reasonable conclusion that can be drawn from considering variance is that the PAS performs in the same range as other measures of its type.

## 7. Future Directions

**Expanding the situations.** The PAS was created using students’ descriptions of two common academic situations—studying and writing—but the construct is not theoretically confined to them. The PAS items reference structural features of preparation and generally minimize content-specific features. Where they do use descriptors that narrow the context, these are often vague references to projects and subjects. The most specific contextual word is “study,” which appears in five questions, but even that term is conceptually broad, since studying can often be used as a synonym for academic work more broadly as long as the context does not specifically indicate studying for an exam.

Because the PAS items emphasize structure rather than the substantive content of writing or studying, it should be possible to adapt them to other academic contexts, including rehearsing for performances, constructing presentations, or other forms of academic preparation. Extending the measure to these is a natural future direction, and a logical step after that would be to develop variants of the PAS for application in non-academic contexts such as the workforce. But—as mentioned above—applying the measure to a new context should be accompanied by validation. An alternative would be to produce a version with even fewer contextual indicators, which could be accomplished with modest rewording, which might produce a “domain general” scale that could be applied flexibly to different activities by describing the scale target in the introductory text rather than altering items—this may be a useful next step.

**The adaptive undercurrent of preparation anxiety**. The suppression effects found in the present study offer an intriguing path forward, as they suggest that preparation anxiety is not completely maladaptive. This echoes historic work on anxiety, which suggests that some arousal is important for performance (Yerkes & Dodson, 1908). While this is known in the anxiety literature as a whole, we would suggest that one of the useful features of our measure is that it provides a possible avenue for studying adaptive effects of anxiety in preparatory contexts. Establishing when (and whom) anxious arousal motivates rather than disrupts would be a valuable contribution to the literature and may help to address longstanding theoretical concerns such as the depressed relationship between anxiety and objective behavioral measures of procrastination (Horton, 2021). Beyond addressing old controversies, we would also suggest that the PAS subscales offer the possibility for testing the suppression effects for moderation by external variables such as gender, personality, or developmental stage: This would allow for questions related to what variables moderate whether a person copes with anxiety adaptively.

**Micro-versus macro-avoidance over time.** As a final note, our analyses support that the PAS-BA, which measures micro-level avoidance, is separable from procrastination. We would suggest, though, that a great deal more research could be done on the relationship between the two. It is not difficult to imagine theoretical cases where a person who procrastinates until the day before the exam, but who does not struggle with in-the-moment avoidance, performs well in spite of their macro-level delay. It is also possible to imagine the opposite—a person who struggles with leaving their chair while working, but who insists on starting and finishing early, might similarly do well. It is also possible that the relationship between the two decreases over time, with micro-level avoidant behaviors decreasing as deadline pressure increases. But these are just conjectures—the degree to which these scenarios exist “in the wild” is not yet established: We believe that the PAS offers researchers an opportunity to explore these distinctions and their relation to student success.

## 8. Conclusions

This article began with the observation that preparation anxiety has received only a modest amount of development in the psychological literature—while it has been addressed indirectly as a function of research on self-sabotage, and while some researchers have attempted to address preparation contexts by appealing to anticipation of future performance, constructs that assess preparation anxiety directly are limited to a few measures developed to assess very specific activities such as writing. In the present article, we sought to address this gap. Across three studies, we developed a 32-item measure of preparation anxiety, the PAS. The measure is multidimensional, capturing affective, behavioral, and cognitive components of students’ struggle with anxiety during preparation. Analysis supports using the measure as a composite and also using its subscales—ideally in tandem, as doing so reveals complex patterns of relationships to academic outcomes. As the measure was built using students’ descriptions of their own struggles, it provides an experientially grounded assessment of what students experience when they sit down to work. Much remains to be done, but the foundation laid here—a psychometrically sound measure of preparation anxiety rooted in students’ lived experience—is a good one to build on. We look forward with anticipation to what future research will bring.

## Figures and Tables

**Table 1:** Interview responses and resulting items.

Initial Statement	Coded Item (from 66-Item Pool)
“Mhm yeah, but there are some times, uh, where I like hyperventilate sometimes so it’s like, yeah. It’s hard to breathe sometimes.”	4. I often feel like I’m having trouble breathing.
“For me, it’s compulsive. For finals I usually delete, like, Instagram and stuff, but like I’ll go on my phone and try to click on it and realize… I think I do it without actually thinking about it.	24. I often find myself automatically clicking on internet sites or browsing social media without intending to.
“Um, sometimes like, I’ll find chores to do that I don’t usually do like clean my room and all that.”	30. I will avoid it by doing chores.
“I’ll just be thinking about it. Like, if I’m not doing it, I’ll be like ‘Oh I need to do it.’ It’s just like, I’m constantly thinking about it…”	39. It is in the back of my mind even when I am not working on the assignment.
“…when it’s like closer to the due date or the exam, and even though I have studied I feel like I haven’t done enough.”	56. I feel as though I could have always done more to prepare.

**Table 2:** Participant Demographics Across Studies.

	Study 2	Study 3 (Full)	Study 3 (Final)
N	252	464	288
Age, M (SD)	19.74 (1.78)	19.42 (1.88)	19.28 (1.50)
***Gender*** **, *n (%)***			
Female	170 (67.5%)	272 (58.6%)	168 (58.3%)
Male	79 (31.3%)	174 (37.5%)	113 (39.2%)
Other	3 (1.2%)	4 (0.9%)	2 (0.7%)
***Race/Ethnicity*** **, *n (%)***			
African American	7 (2.8%)	10 (2.2%)	6 (2.1%)
Asian/Pacific Islander	105 (41.7%)	171 (36.9%)	116 (40.3%)
Caucasian	24 (9.5%)	30 (6.5%)	13 (4.5%)
Hispanic/Latinx	84 (33.3%)	178 (38.4%)	105 (36.5%)
Middle Eastern	19 (7.5%)	21 (4.5%)	13 (4.5%)
Native American	—	3 (0.6%)	2 (0.7%)
Multiracial/Other	12 (4.8%)	37 (8.0%)	28 (9.7%)
***Class Standing*** **, *n (%)***			
Freshman	79 (31.3%)	233 (50.2%)	141 (49.0%)
Sophomore	80 (31.7%)	106 (22.8%)	77 (26.7%)
Junior	47 (18.7%)	83 (17.9%)	49 (17.0%)
Senior	45 (17.9%)	27 (5.8%)	15 (5.2%)
***Major Category*** **, *n (%)***			
STEM	116 (46.0%)	177 (38.1%)	120 (41.7%)
HASS	107 (42.5%)	231 (49.8%)	133 (46.2%)
Undeclared	24 (9.5%)	37 (8.0%)	26 (9.0%)

*Note.* Study 2 (*N* = 252) and Study 3 are separate samples. Study 3 survey took two sessions to complete; the PAS was administered in the second. The Study 3 (Final) sample (*N* = 288) consists of those who filled out the PAS and was used for analysis. The Study 3 (Full) sample (*N* = 464) is reported for transparency. STEM = Science, Technology, Engineering, Math. HASS = Humanities, Arts, Social Sciences (category subsumes business and administration). Blanks not reported: Percentages may sum to less than 100%.

**Table 3:** Full List of Measures Used to Assess Convergent and Discriminant Validity.

Measure	Citation	Format	*α (S2)*	*α (S3)*
Self-Handicapping Scale (SHS)	(Jones & Rhodewalt, 1982)	25 items, 6-point	0.74	
Academic Self-Handicapping Scale (ASHS)	(Midgley & Urdan, 1995)	6 items, 5-point	0.81	
Beck Depression Inventory-II (BDI)	(Beck et al., 1996)	21 items, 0–3 scale	0.92	
State-Trait Anxiety Inventory (STAI)—Trait	(Spielberger et al., 1983)	20 items, 4-point	0.93	
TOSCA-3 Short—Shame subscale	(Tangney et al., 1989)	Scenario-based	0.77	
TOSCA-3 Short—Guilt subscale	(Tangney et al., 1989)	Scenario-based	0.68	
Rosenberg Self-Esteem Scale (RSES)	(Rosenberg, 1965)	10 items, 4-point	0.90	
Sarason Test Anxiety Scale (STAS)	(Sarason, 1980)	37 items, True/False	0.89	
Westside Test Anxiety Scale (WTAS)	(Driscoll, 2007)	10 items, 5-point		0.89
DASS-21—Anxiety subscale	(Lovibond & Lovibond, 1995)	7 items, 4-point		0.84
Big Five Inventory (BFI)	(John et al., 2008) ***(S2)*** (Soto & John, 2017) (S3) *	44 items, 5-point 42 items, 5-point		
Openness			0.74	0.70
Conscientiousness			0.65	0.85
Extraversion			0.86	0.77
Agreeableness			0.63	0.73
Neuroticism			0.85	0.88
Pure Procrastination Scale (PPS)	(Steel, 2010)	12 items, 5-point	0.93	0.90
Intolerance of Uncertainty Scale (IOU)	(Buhr & Dugas, 2002)	12 items, 5-point	0.94	
Self-Liking/Self-Competence Scale (SLCS-R)	(Tafarodi & Swann, 2001)	16 items, 5-point	0.94	
Self-Liking			0.91	
Self-Competence			0.67	
Academic Self-Efficacy Scale (ASE)	(Chemers et al., 2001)	19 items, 7-point		0.94
Physical Health Questionnaire (PHQ)	(Schat et al., 2005)	14 items, 7-point		0.75

*Note.* α = Cronbach’s alpha. S2 = Study 2; S3 = Study 3. Blank cells indicate the measure was not administered in that study. Subscales are indented beneath their parent measure. * The 60-item BFI-2 from Soto and John (2017) was truncated in Study 3: Openness, Extraversion, and Agreeableness were cut to 6 items, while Neuroticism and Conscientiousness were kept at 12.

**Table 4:** Standardized Factor Loadings for the 32-Item PAS from Exploratory (Study 2) and Confirmatory (Study 3) Factor Analyses of the Preparation Anxiety Scale.

Item	Item Text	*EFA λ*	*CFA λ*
	***Factor 1: Behavioral Avoidance (Study 2 α = 0.92*** **, *Study 3 α/ω = 0.90/0.91)***		
31	I make excuses not to study.	0.86	0.70
22	I find it difficult to focus.	0.73	0.77
30	I will avoid it by doing chores.	0.73	0.49
28	I can’t help but get distracted by more trivial things.	0.71	0.71
49	I work on unimportant assignments first.	0.69	0.56
23	I feel like I really have to force myself to do it.	0.68	0.81
48	I have a lot of trouble just getting started.	0.66	0.72
51	I feel very confused at first.	0.63	0.63
24	I often find myself automatically clicking on internet sites or browsing social media without intending to.	0.62	0.72
58	I am able to focus easily. ^R^	0.57	0.56
65	I spend more time worrying about my schoolwork than I do working on it.	0.55	0.78
20	I spend more time organizing myself to study than actually studying.	0.49	0.59
52	I get upset with myself for not studying sooner.	0.47	0.79
40	I have no trouble motivating. ^R^	0.44	0.47
59	I feel like I just can’t do it right.	0.42	0.72
	***Factor 2: Cognitive Agitation (Study 2 α = 0.89*** **, *Study 3 α/ω = 0.85/0.86)***		
19	I worry about the uncertainty of my grade.	0.82	0.76
15	I feel frustrated with myself when I am not able to focus.	0.74	0.85
14	I get frustrated when I feel I am not studying effectively.	0.73	0.75
56	I feel as though I could have always done more to prepare.	0.67	0.74
38	I am not worried about my grade. ^R^	0.64	0.35
36	I worry about failing the exam or project.	0.62	0.76
43	I get frustrated when I do not know what I should be studying.	0.57	0.56
26	I have a lot of trouble focusing when it is a subject I do not like.	0.56	0.74
62	I get nervous when it takes longer than expected to finish.	0.42	0.77
	***Factor 3: Affective Nerves (Study 2 α = 0.87*** **, *Study 3 α/ω = 0.89/0.89)***		
8	I need to calm myself down frequently.	0.87	0.77
9	I feel very nervous/anxious.	0.84	0.89
11	When I look back on my behavior while working on a project, I often realize that I must have been anxious or stressed, even if I didn’t know it at the time.	0.65	0.74
17	I feel like my thoughts start racing.	0.61	0.67
7	I can’t get it out of my mind even when I’m away from it.	0.58	0.65
50	I feel like my emotions get out of hand.	0.57	0.77
10	I tend to lose sleep.	0.53	0.67
66	I feel discomfort.	0.47	0.83

*Note.* EFA λ = promax-rotated pattern coefficient (principal axis factoring, *N* = 252). CFA λ = standardized loading (WLSMV estimator, *N* = 288). ^R^ = reverse-scored item. Items ordered by EFA loading magnitude within each factor. PAS items were rated on a 5-point Likert scale (1 = Strongly Disagree to 5 = Strongly Agree). Item pas.40 (“I have no trouble motivating”) is an incomplete sentence: we have reported it as administered in the study for transparency. Researchers may wish to use the complete version: “I have no trouble motivating myself.”.

**Table 5:** Descriptive Statistics and Bivariate Correlations Among Study Variables (Study 2, N = 252).

	*M*	*SD*	1	2	3	4	5	6	7	8	9	10	11	12	13	14	15	16	17	18	19
1. PAS-Total	3.58	0.69	—																		
2. PAS-BA	3.48	0.80	**0.92**	—																	
3. PAS-CA	3.94	0.74	**0.83**	**0.64**	—																
4. PAS-AN	3.35	0.85	**0.81**	**0.60**	**0.59**	—															
5. SHS	3.51	0.51	**0.63**	**0.67**	**0.36**	**0.50**	—														
6. ASHS	1.83	0.76	**0.22**	**0.27**	0.04	**0.19**	**0.35**	—													
7. BDI	0.70	0.48	**0.62**	**0.57**	**0.45**	**0.56**	**0.61**	**0.28**	—												
8. TOSCA-S	3.31	0.66	**0.53**	**0.42**	**0.47**	**0.52**	**0.34**	0.11	**0.38**	—											
9. TOSCA-G	4.19	0.56	**0.21**	0.08	**0.28**	**0.24**	−0.01	**−0.22**	0.09	**0.41**	—										
10. STAI	2.51	0.56	**0.71**	**0.64**	**0.55**	**0.63**	**0.67**	**0.30**	**0.80**	**0.44**	0.11	—									
11. RSES	2.37	0.59	**0.64**	**0.60**	**0.46**	**0.56**	**0.63**	**0.25**	**0.73**	**0.42**	0.04	**0.85**	—								
12. STAS	24.65	7.55	**0.61**	**0.49**	**0.51**	**0.62**	**0.42**	**0.15**	**0.50**	**0.45**	**0.29**	**0.55**	**0.47**	—							
13. BFI-E	2.83	0.79	**−0.27**	**−0.31**	**−0.15**	**−0.19**	**−0.38**	−0.12	**−0.41**	**−0.19**	−0.03	**−0.45**	**−0.44**	**−0.23**	—						
14. BFI-A	3.61	0.54	−0.09	**−0.15**	0.01	−0.02	**−0.20**	−0.10	**−0.24**	0.00	**0.36**	**−0.26**	**−0.30**	0.09	**0.15**	—					
15. BFI-C	3.09	0.52	**−0.42**	**−0.53**	**−0.18**	**−0.27**	**−0.61**	**−0.24**	**−0.42**	**−0.20**	0.12	**−0.42**	**−0.49**	**−0.14**	**0.31**	**0.34**	—				
16. BFI-N	3.33	0.79	**0.68**	**0.56**	**0.55**	**0.69**	**0.60**	**0.17**	**0.68**	**0.51**	**0.18**	**0.77**	**0.69**	**0.60**	**−0.37**	**−0.15**	**−0.31**	—			
17. BFI-O	3.37	0.58	−0.11	**−0.21**	0.02	−0.01	**−0.15**	**−0.15**	**−0.14**	0.01	0.11	**−0.13**	**−0.22**	−0.10	**0.29**	**0.15**	**0.19**	−0.04	—		
18. PPS	2.96	0.97	**0.62**	**0.70**	**0.32**	**0.47**	**0.66**	**0.43**	**0.50**	**0.34**	−0.04	**0.53**	**0.51**	**0.34**	**−0.32**	**−0.18**	**−0.63**	**0.44**	**−0.23**	—	
19. IOU	3.05	0.75	**0.55**	**0.47**	**0.40**	**0.55**	**0.46**	**0.18**	**0.56**	**0.46**	0.07	**0.59**	**0.58**	**0.45**	**−0.34**	**−0.18**	**−0.33**	**0.55**	−0.07	**0.46**	—

Note. n = 252. Boldface coefficients are significant at *p* < 0.05; as a guide, |r| ≥ 0.12, ≥0.16, and ≥0.21 correspond to *p* < 0.05, 0.01, and 0.001, respectively. PAS-Total = Preparation Anxiety; PAS-BA = Behavioral Avoidance subscale; PAS-CA = Cognitive Agitation subscale; PAS-AN = Affective Nerves subscale; SHS = Self-Handicapping Scale; ASHS = Academic Self-Handicapping Scale; BDI = Beck Depression Inventory-II; TOSCA-S/TOSCA-G = TOSCA-3 Shame and Guilt subscales; STAI = State-Trait Anxiety Inventory (Trait); RSES = Rosenberg Self-Esteem Scale; STAS = Sarason Test Anxiety Scale; BFI = Big Five Inventory (E = Extraversion, A = Agreeableness, C = Conscientiousness, N = Neuroticism, O = Openness); PPS = Pure Procrastination Scale; IOU = Intolerance of Uncertainty Scale.

**Table 6:** Confirmatory Factor Analysis Model Comparison (Study 3, N = 288).

Estimator	Model	χ^2^	df	CFI	TLI	RMSEA [90% CI]	SRMR	Δχ^2^ (vs.)
WLSMV	1-Factor	2340.68	464	0.791	0.777	0.119 [0.114, 0.124]	0.101	—
WLSMV	3-Factor (Correlated)	1489.62	461	0.886	0.877	0.088 [0.083, 0.093]	0.077	278.20 *** (vs. 1-F)
WLSMV	Bifactor (Symmetric)	1190.45	432	0.916	0.903	0.078 [0.073, 0.084]	0.065	782.03 *** (vs. 1-F)
WLSMV	Bifactor (+4 Resid.)	1054.42	428	0.930	0.919	0.071 [0.066, 0.077]	0.061	146.05 *** (vs. BF)
MLR	1-Factor	1665.25	464	0.691	0.670	0.101 [0.096, 0.107]	0.084	—
MLR	3-Factor (Correlated)	1132.20	461	0.829	0.816	0.076 [0.070, 0.081]	0.071	261.40 *** (vs. 1-F)
MLR	Bifactor (Symmetric)	987.90	432	0.872	0.853	0.068 [0.062, 0.074]	0.063	401.52 *** (vs. 1-F)
MLR	Bifactor (+4 Resid.)	842.58	428	0.900	0.884	0.060 [0.054, 0.066]	0.055	— (vs. BF)

Note. χ^2^ are robust/scaled statistics (WLSMV scaled; MLR Yuan–Bentler robust); CFI/TLI/RMSEA likewise. Scaled χ^2^ are not additive across models; Δχ^2^ are scaled difference tests (Satorra & Bentler, 2001), each against the noted nested model. The bifactor and three-factor models are not nested and are not compared by χ^2^ difference. *** *p* < 0.001.

**Table 7:** Bifactor Reliability and Dimensionality Indices (Study 3, N = 288).

Factor	ω	ωH/ωHS	ECV	H	PUC
General (PA)	0.963	0.845	0.689	0.952	0.659
PAS-BA	0.929	0.192	0.259	0.706	
PAS-CA	0.899	0.270	0.321	0.660	
PAS-AN	0.913	0.332	0.383	0.725	

Note. From the standardized bifactor solution with four freed residual covariances (WLSMV). ω = total reliability; ωH = omega-hierarchical (general); ωHS = omega-hierarchical-subscale; ECV = explained common variance; H = construct replicability; PUC = proportion of uncontaminated correlations.

**Table 8:** Descriptive Statistics and Bivariate Correlations Among Study Variables (Study 3, N = 288).

Variable	M	SD	1	2	3	4	5	6	7	8	9	10	11	12	13	14	15
1. PAS-Total	3.47	0.67	—														
2. PAS-BA	3.36	0.75	**0.92**	—													
3. PAS-CA	3.90	0.70	**0.80**	**0.62**	—												
4. PAS-AN	3.18	0.92	**0.83**	**0.63**	**0.53**	—											
5. GPA (Overall)	3.32	0.47	−0.11	**−0.17**	−0.04	−0.03	—										
6. GPA (Major)	3.35	0.49	**−0.14**	**−0.19**	−0.08	−0.05	**0.90**	—									
7. PHQ	4.42	1.31	**0.32**	**0.26**	**0.16**	**0.40**	−0.02	−0.06	—								
8. PPS	2.96	0.76	**0.55**	**0.63**	**0.29**	**0.40**	**−0.16**	**−0.18**	**0.21**	—							
9. ASE	7.64	1.89	**−0.35**	**−0.40**	**−0.16**	**−0.29**	**0.18**	**0.23**	**−0.16**	**−0.44**	—						
10. WTAS	3.20	0.84	**0.44**	**0.41**	**0.32**	**0.38**	**−0.16**	**−0.24**	**0.22**	**0.38**	**−0.26**	—					
11. DASS-Anx	11.62	4.35	**0.43**	**0.34**	**0.18**	**0.57**	−0.07	−0.04	**0.40**	**0.30**	**−0.17**	**0.17**	—				
12. BFI-N	3.06	0.77	**0.62**	**0.54**	**0.42**	**0.62**	−0.09	−0.10	**0.38**	**0.48**	**−0.36**	**0.33**	**0.43**	—			
13. BFI-C	3.38	0.66	**−0.33**	**−0.41**	**−0.14**	**−0.22**	**0.18**	**0.17**	−0.03	**−0.61**	**0.43**	**−0.13**	**−0.13**	**−0.41**	—		
14. BFI-A	3.71	0.69	−0.11	**−0.15**	0.01	−0.09	−0.00	−0.01	−0.09	**−0.22**	**0.18**	−0.05	**−0.12**	**−0.28**	**0.42**	—	
15. BFI-O	3.62	0.68	−0.11	**−0.20**	−0.02	−0.01	0.10	**0.15**	0.02	**−0.24**	**0.24**	−0.11	0.03	**−0.14**	**0.22**	0.08	—
16. BFI-E	3.11	0.80	**−0.19**	**−0.23**	**−0.16**	−0.08	0.09	0.09	0.10	**−0.23**	**0.15**	−0.06	0.04	**−0.35**	**0.26**	0.10	**0.22**

Note. n = 288. Boldface coefficients are significant at *p* < 0.05; as a guide, |r| ≥ 0.12, ≥0.15, and ≥0.19 correspond to *p* < 0.05, 0.01, and 0.001, respectively (GPA correlations use reduced n: 258 overall, 221 major). DASS-Anx is the 7-item sum; other multi-item variables are item means; WTAS is scored so higher = more test anxiety. PAS-BA/CA/AN = Behavioral Avoidance/Cognitive Agitation/Affective Nerves; PHQ = Physical Health Questionnaire; PPS = Pure Procrastination Scale; ASE = Academic Self-Efficacy; WTAS = Westside Test Anxiety Scale; DASS-Anx = DASS-21 Anxiety; BFI = Big Five Inventory (N/C/A/O/E).

**Table 9:** Standardized Regression Models Predicting Criterion Variables from PAS Subscales (Study 3).

Predictor	β	SE	t	*p*	R^2^
**Procrastination (PPS) (N = 287)**					**0.411**
PAS-BA	0.712	0.065	10.897	0.000 ***	
PAS-CA	−0.180	0.060	−2.999	0.003 **	
PAS-AN	0.042	0.060	0.691	0.490	
**Academic Self-Efficacy (ASE) (N = 283)**					**0.183**
PAS-BA	−0.451	0.077	−5.826	0.000 ***	
PAS-CA	0.178	0.071	2.496	0.013 *	
PAS-AN	−0.100	0.072	−1.390	0.166	
**Physical Health (PHQ) (N = 283)**					**0.164**
PAS-BA	0.063	0.078	0.811	0.418	
PAS-CA	−0.102	0.072	−1.408	0.160	
PAS-AN	0.412	0.073	5.681	0.000 ***	
**Overall GPA (N = 258)**					**0.045**
PAS-BA	−0.295	0.087	−3.398	0.001 ***	
PAS-CA	0.099	0.083	1.191	0.235	
PAS-AN	0.098	0.080	1.234	0.218	
**Major GPA (N = 221)**					**0.043**
PAS-BA	−0.261	0.092	−2.848	0.005 **	
PAS-CA	0.033	0.089	0.371	0.711	
PAS-AN	0.087	0.085	1.021	0.308	

Note. Bolded rows report criterion variable, sample size. Each block is a multiple regression of the criterion on PAS-BA, PAS-CA, PAS-AN. β = Standardized betas. * *p* < 0.05, ** *p* < 0.01, *** *p* < 0.001.

**Table 10:** Standardized Regression Models Predicting Criterion from PAS Subscales and Competing Measures (Study 3).

Model	PAS-BA	PAS-CA	PAS-AN	WTAS	DASS-A	BFI-N	R^2^
**Panel A: Procrastination (PPS)**							
PAS only	0.712 ***	−0.180 **	0.042	—	—	—	0.411
+ WTAS	0.671 ***	−0.189 **	0.012	0.159 **	—	—	0.432
+ DASS-A	0.706 ***	−0.159 **	−0.027	—	0.106	—	0.418
+ BFI-N	0.654 ***	−0.187 **	−0.073	—	—	0.249 ***	0.447
+ All three	0.617 ***	−0.178 **	−0.143 *	0.149 **	0.083	0.224 ***	0.469
Competitors only	—	—	—	0.246 ***	0.108	0.351 ***	0.295
**ΔR^2^ (PAS beyond competitors)**							**0.174**
**Panel B: Academic Self-Efficacy (ASE)**							
PAS only	−0.451 ***	0.178 *	−0.100	—	—	—	0.183
+ WTAS	−0.420 ***	0.186 **	−0.078	−0.122 *	—	—	0.195
+ DASS-A	−0.452 ***	0.179 *	−0.104	—	0.007	—	0.183
+ BFI-N	−0.402 ***	0.185 **	−0.000	—	—	−0.218 **	0.210
+ All three	−0.377 ***	0.198 **	−0.002	−0.108	0.029	−0.213 **	0.221
Competitors only	—	—	—	−0.165 **	−0.021	−0.294 ***	0.151
**ΔR^2^ (PAS beyond competitors)**							**0.069**
**Panel C: Physical Health (PHQ)**							
PAS only	0.063	−0.102	0.412 ***	—	—	—	0.164
+ WTAS	0.042	−0.107	0.397 ***	0.086	—	—	0.170
+ DASS-A	0.049	−0.052	0.255 **	—	0.243 ***	—	0.202
+ BFI-N	0.012	−0.109	0.308 ***	—	—	0.228 **	0.195
+ All three	−0.014	−0.067	0.167	0.083	0.222 ***	0.190 **	0.230
Competitors only	—	—	—	0.096	0.283 ***	0.227 ***	0.219
**ΔR^2^ (PAS beyond competitors)**							**0.011**
**Panel D: Overall GPA**							
PAS only	−0.295 ***	0.099	0.098	—	—	—	0.045
+ WTAS	−0.259 **	0.109	0.121	−0.134	—	—	0.059
+ DASS-A	−0.291 ***	0.084	0.143	—	−0.070	—	0.048
+ BFI-N	−0.282 **	0.098	0.121	—	—	−0.052	0.046
+ All three	−0.248 **	0.094	0.177	−0.134	−0.069	−0.027	0.063
Competitors only	—	—	—	−0.147 *	−0.027	−0.028	0.028
**ΔR^2^ (PAS beyond competitors)**							**0.035**
**Panel E: Major GPA**							
PAS only	−0.261 **	0.033	0.087	—	—	—	0.043
+ WTAS	−0.198 *	0.052	0.122	−0.222 **	—	—	0.081
+ DASS-A	−0.261 **	0.030	0.099	—	−0.018	—	0.043
+ BFI-N	−0.250 **	0.033	0.113	—	—	−0.055	0.045
+ All three	−0.191 *	0.048	0.150	−0.220 **	−0.023	−0.030	0.082
Competitors only	—	—	—	−0.237 ***	0.015	−0.027	0.060
**ΔR^2^ (PAS beyond competitors)**							**0.023**

Note. Standardized betas; each panel one criterion. Titles and ΔR^2^ rows bolded. ΔR^2^ = increment for PAS subscales over competing measures (WTAS, DASS, BFI-N). * *p* < 0.05, ** *p* < 0.01, *** *p* < 0.001.

## Data Availability

Data has not been made public due to privacy restrictions, but may be made available upon request from the authors.
